# Understanding the Extent of Polypharmacy and its Association With Health Service Utilization Among Persons With Cancer and Multimorbidity: A Population-Based Retrospective Cohort Study in Ontario, Canada

**DOI:** 10.1177/08971900221117105

**Published:** 2022-07-21

**Authors:** Tamara Dean, Anna Koné, Lynn Martin, Joshua Armstrong, Caroline Sirois

**Affiliations:** 1 Department of Health Sciences, 7890 Lakehead University, Thunder Bay, ON, Canada; 2 Faculté de pharmacie, 4440 Université Laval, Quebec City, QC, Canada

**Keywords:** polypharmacy, cancer, multimorbidity, health service utilization, adverse drug events

## Abstract

**Background:** Cancer often co-occurs with other chronic conditions, which may result in polypharmacy. Polypharmacy is associated with adverse outcomes, including increased health service utilization. **Objectives:** This study examines the overall prevalence of polypharmacy (5 or more medications) among adults with cancer and multimorbidity, as well as the association of both minor polypharmacy (5-9 medications) and hyper-polypharmacy (10 or more medications) on high use of emergency room visits and hospitalizations, while controlling for age, sex, and type and stage of cancer. **Methods:** This retrospective longitudinal study used linked health administrative databases and included persons 18 years and older diagnosed with cancer between April 2010 and March 2013 in Ontario, Canada. Data on the number of health service utilizations at or above the 90th percentile (high users), was collected up to March 2014 and multivariate logistic regression was used to determine the impact of polypharmacy. **Results:** The prevalence of polypharmacy was 46% prior to cancer diagnosis, and 57% one year after diagnosis. Polypharmacy prior to and after cancer diagnosis increased with the level of multimorbidity, increasing age, but did not differ by sex. It was also highest in persons with lung cancer (52.4%) and those diagnosed with stage 4 cancer (51.3%). Minor polypharmacy increased the odds of being a high user of emergency rooms (1.16; 99% CI: 1.09-1.24) and hospitalizations (1.03; 0.98-1.09) and the odds of high use was greater with hyper-polypharmacy (1.41; 1.33-1.51) and (1.23; 1.17-1.29) respectively. **Conclusion:** Polypharmacy is highly prevalent and is associated with high health service utilization among adults with cancer and multimorbidity

## Introduction

Cancer is the second leading cause of mortality worldwide^1,2^ and the burden of cancer is increasing globally, as a result of varied at-risk behaviours and the growth of an aging population.^3^ According to the Canadian Cancer Statistics^4^ one in two Canadians will be diagnosed with cancer in their lifetime. The risk of cancer increases with age, where 90% of newly diagnosed cases will consists of Canadians over the age of 50 years.^4^

The prevalence of multimorbidity, defined as the co-occurrence of two or more chronic health conditions, it is very common among older persons,^5^ and is increasing among younger adults.^6,7^ Cancer is also considered a chronic disease that is prevalent in the elderly, and therefore multimorbidity among persons with cancer is common.^8^ A challenge related to multimorbidity is polypharmacy, as each co-occurring condition may require different therapeutic approaches.

Polypharmacy has various definitions, it is defined by numerical groupings with associated terms such as hyper polypharmacy or excessive polypharmacy,^9-12^ or by a descriptive definition such as appropriate or inappropriate polypharmacy.^11,13^ In the literature the most common definition is the use of five or more drugs.^10,11,14^ Polypharmacy may be a consequence of both cancer^15^ and multimorbidity.^16^ Not surprisingly, persons with cancer and multimorbidity have an increased number of medications prescribed.^11^ For example, a more advance cancer disease is linked to an even higher number of medications.^17^

Other factors associated with polypharmacy include age and sex.^18,19^ In Canada approximately one-quarter of elderly people are prescribed 10 or more medications over a year.^20^ Being female is also a risk factor for polypharmacy, as is the combination of being female and age 85 years and older.^21,22^

Pharmacotherapy plays an integral role in cancer treatment. However, the risk of adverse drug events increases as a result of increased number of prescribed medications.^21^ The added adverse events incurred by polypharmacy can lead to higher health service utilization, such as emergency room visits and hospitalizations, and death, especially in the elderly.^16,23-25^

The prevalence of polypharmacy and the associated negative health outcomes among older adults with cancer and multimorbidity has been extensively discussed in the literature, however the prevalence and impact of polypharmacy among young adults with cancer and other chronic diseases are not adequately represented in the literature. Thus, this study examines whether the prevalence of polypharmacy differs by age, sex, level of multimorbidity and type and stage of cancer among both young and older adults. In addition, it assesses the relationship between polypharmacy and health service utilization (ie, emergency room visits and hospitalizations) in the year following cancer diagnosis.

## Ethical Approval

Ethics approval was obtained from Lakehead University Research Ethics Board and the Institute for Clinical Evaluative Services (ICES), who provided access to the data.

## Method

### Study Design and Population

This study used a retrospective longitudinal design linking health administrative databases. This study included Ontarians 18 years and older with a valid Ontario Health Insurance Plan (OHIP) card, diagnosed with cancer between April 1, 2010 and March 31, 2013 (they may have been diagnosed with one of the selected chronic conditions prior to the cancer diagnosis). Our study population consists of persons with and without Ontario Drug Benefit (ODB) program coverage for the entire follow-up period (one year post-diagnosis). ODB program recipients include all Ontarians 65 years or older. Ontarians who are under the age of 65 will qualify for the ODB program if they are residents of long-term care facilities or homes for special care, persons receiving services under the Home Care Program, Trillium Drug Program recipients, persons receiving special assistance (eg, Ontario Works, Ontario Disability Support Program), and persons who are eligible for the Special Drugs Program (SDP), regardless of age. It was only as of January 1, 2018, that all persons 24 years and younger who were not covered by a private insurance plan also qualified for the ODB program.^26^ Different data sources were used, and eligibility for each program (eg, Ontario Drug Benefit Program) is defined by provincial rules regarding health coverage. All individuals covered by OHIP are eligible for physician, primary care, hospital and emergency services, and thus are included in OHIP physicians’ claims, Discharge Abstract Database (DAD), and National Ambulatory Care Reporting System (NACRS). People over 65 as well as a portion of those <65 (as described above) are eligible for medication coverage and included in ODB. Persons who died within one year of their cancer diagnosis were excluded from the study. These persons may have had more severe disease or complications of their chronic diseases and cancer and were excluded to avoid bias and to minimize reverse causality.^27,28^

Follow-up data was used to assess service utilization within one year after diagnosis.

### Data Sources

The data in this study were obtained from de-identified linked health databases securely housed at the Institute for Clinical Evaluative Services (ICES) and remotely accessed through their Data and Analytic Virtual Environment (see Table 1). ICES utilized encoded identifiers to generate the study population, to determine the occurrence of chronic conditions, and to quantify health service utilization.^29,30^

**Table 1.** Institute for Clinical Evaluative Services Databases That Provided Data for the Study.

Database	Description
National ambulatory care reporting system (NACRS)	Information on outpatient hospital visits and community based ambulatory care (physician office visits). It includes day surgery, emergency room visits, and outpatient clinics
Discharge abstract database (DAD)	Information on hospitalizations in Ontario, including admissions, length of hospital stays and discharges
Ontario drug benefit (ODB) claims database	Information about drug claims by ODB beneficiaries, such as *drug identification number*, number of drugs dispensed, and date dispensed
Registered person database files (RPDB)	Demographic information (eg, sex, age, neighbourhood income, rural/urban residence) for all persons who have ever been issued a valid Ontario health card number
Ontario health insurance plan (OHIP) claims database	A health plan ran by the government of Ontario, provides data on claims paid for by OHIP, including hospitalizations and associated diagnosis, physician visits, medications prescribed while in hospital and some medications in the ambulatory setting
Ontario cancer registry (OCR), database	Information on all persons living in Ontario who were diagnosed with cancer, that will include date of diagnosis, site of primary cancer and date of cancer death

Approaches to identifying similar study cohorts and chronic conditions from provincial datasets are presented in previous studies.^30,31^ The list of ICD codes used to identify these chronic conditions is provided in Supplementary Appendix A.

### Variables

The definition and operationalization of all variables (ie, polypharmacy, health services utilization, multimorbidity, age, sex, cancer type and stage), are summarized in Table 2. Note that “prior polypharmacy” refers to the number of drugs prescribed within the year prior to cancer diagnosis, while “polypharmacy post diagnosis” refers to those prescribed up to one year after diagnosis. For each, polypharmacy was defined as the use of five or more drugs^11^ and was categorized into three groups: <5 drugs (no polypharmacy), 5-9 (minor polypharmacy), and 10 or more drugs (hyper-polypharmacy). All prescribed drugs were associated with a drug identification number (DIN), which was assigned by Health Canada.^32^ The number of emergency room visits and hospitalizations in the year following cancer diagnosis was examined to determine the number equalling the 90th percentile. The number of emergency room visits and hospital admissions per person-year was then categorized to reflect “high-use” of health services, based on being at or above the 90th percentile.

**Table 2.** Description of Variables (All are Categorical).

Variable	Value
Polypharmacy prior (using the number of drugs prescribed within the year prior to cancer diagnosis)	<5 = none
	5-9 = minor polypharmacy
	10+ = hyper-polypharmacy
Polypharmacy after (using the number of drugs prescribed one year post cancer diagnosis; excludes chemotherapy drugs)	<5 = none
	5-9 = minor polypharmacy
	10+ = hyper-polypharmacy
Hospitalizations within one year post-diagnosis	1 = high user (≥90th percentile)
	0 = not a high user
Emergency room visits within one year post-diagnosis	1 = high user (≥ 90th percentile)
	0 = not a high user
Age group at the time of cancer diagnosis	1 = ≥18 years to 44 years
	2 = 45 years to 64 years
	3 = ≥65 years
Sex	Male/female
Multimorbidity (among 17 conditions: Acute myocardial infarction, chronic coronary syndrome, congestive heart failure, chronic obstructive pulmonary disease, diabetes mellitus, hypertension, asthma, stroke, osteoporosis, dementia, arrhythmias, rheumatoid arthritis, osteoarthritis, anxiety, renal failure, psychotic and mood disorders)	0 conditions
	1 condition
	2 conditions
	3 or more conditions
Cancer type	Breast, lung and bronchus, female reproductive system, urinary system, colorectal cancer, prostate, digestive system, and other
Cancer stage The stages are measured categorically as; I (the tumor is small and has not grown outside of the primary organ), II and III (the tumor is larger than stage I, or it may have grown outside the primary organ into nearby tissue), and IV (the cancer has spread to a distant site in the body via the blood and lymphatic system) Canadian Cancer Society (n.d.)	I, II, III, IV, unknown
Chemotherapy drugs (number of intravenous and oral antineoplastic drugs prescribed within the year after cancer diagnosis)	<5, 5-9, 10 or more drugs

### Data Analysis

Descriptive statistics (%, mean, SD) characterize the study population for all considered variables. Bivariate analysis (i.e., chi-square test) was used to determine if polypharmacy (pre and post cancer diagnosis) differs by age, sex, multimorbidity, type of cancer, and stage of cancer. Stratified crosstables and chi-square tests inform on the interactions between multimorbidity level, sex, age, and polypharmacy post cancer diagnosis, while a multivariate model was created adjusting for type and stage of cancer. Next, multivariate logistic regression was used to quantify the adjusted relationship between polypharmacy and health service utilization. All data analyses were performed using Stata version 16.1.^33^ Due to large population sample size, level of significance was fixed at *P* < .0001.

## Results

### Study Population Characteristics

The study population consisted of 193 047 Ontarians, with almost 85 000 individuals under 65 (Table 3). The mean age was 65.8 years (sd 14.5); males were slightly older than females (66.8 years vs 64.7 years); 56% of the cohort were 65 years and older, and less than 10% were under the age of 45 years. Overall, cancers of the breast, lung and bronchus, and prostate were most prevalent. Stage of cancer was unknown for more than half of the study population, and among those with known stage, more persons had stage I cancer. About half of the population had three or more chronic conditions.

**Table 3.** Study Population Characteristics (Adults ≥18 Years Who are Eligible for Drug Coverage).

Variables	All	Sex		Age		
		Males	Females	18-44 years	45-64 years	65+ years
	N = 193 047	N = 96 283	N = 96 764	N = 15 039	N = 70 719	N = 107 289
Sex						
Males^a^ (%)	49.9	—	—	35.5	47.1	52
Females^a^ (%)	50.1	—	—	64.5	52.9	48
Age						
18-44 years^b^ (%)	7.8	5.5	10.1	—	—	—
45-64 years^b^ (%)	36.6	35.2	38	—	—	—
65+ years^b^ (%)	55.6	59.3	51.9	—	—	—
Mean (SD)	65.8 (14.5)	66.8 (13.4)	64.7 (15.4)	—	—	—
Cancer type						
Breast cancer^a,b^ (%)	14.1	.2	27.9	18	18	10.9
Colon & rectum^a,b^ (%)	11.3	12	10.5	6.1	9.7	13
Digestive system^a,b^ (%)	8.6	10	7.2	4.1	7.9	9.7
Female genital^a,b^ (%)	6.2	0	12.3	8.7	8	4.6
Hematological^a,b^ (%)	9.3	10.3	8.4	12.4	8	9.8
Lung & bronchus^a^ (%)	12.4	12.8	12	1.8	9.7	15.6
Prostate^a,b^ (%)	13.3	26.7	0	.55	14.3	14.5
Urinary system^a,b^ (%)	6	8.3	3.6	3.2	5.2	6.9
Other^a,b^ (%)	19	19.7	18.2	45.2	19.3	15.1
Cancer stage^a,b^						
Stage I (%)	16.7	10.2	23.2	26.2	19.5	13.5
Stage II (%)	18.6	22.2	15	14.2	20.1	18.3
Stage III (%)	8.9	10.8	11.3	9.6	12.5	10.3
Stage IV (%)	10.9	15.6	12.1	6.5	13.5	15
Unknown (%)	54.2	41.2	38.4	43.4	34.4	42.8
Multimorbidity before cancer^a^						
No condition (%)	13.7	13.6	13.7	40.4	20.1	5.6
1 condition (%)	20.3	20.3	20.3	33.8	27	13.9
2 conditions (%)	21.2	21.1	21.3	16.6	23	20.7
3+ conditions (%)	44.9	45	44.7	9.1	29.9	59.8
Chemotherapy drugs^a,b^	6.5 (12.3)	5.8 (12.1)	7.1 (12.4)	10.6 (16.5)	7.6 (14.1)	5.7 (10.7)
Mean (SD)						
<5 (%)	83.2	86.5	79.9	84	84.6	82.1
5-9 (%)	8.1	6.6	9.6	6	6.5	9.5
10 or more drugs (%)	8.7	6.9	10.5	10	8.9	8.4

^a^Statistically significant difference (*P* < .0001) by age.

^b^Statistically significant difference (*P* < .0001) by sex.

### Polypharmacy

Prior to cancer diagnosis the prevalence of minor polypharmacy and hyper-polypharmacy was 20.9% and 25.1% respectively. Polypharmacy increased after cancer diagnosis, the prevalence of minor polypharmacy slightly decreased after cancer diagnosis (to 18.9%), and hyper-polypharmacy greatly increased (to 37.8%). The decrease in minor polypharmacy was mainly driven by the increase in hyper-polypharmacy (ie, people were more likely to have hyper-polypharmacy) (Table 4). Minor polypharmacy was slightly higher among males at both times and was more prevalent with increasing age. The prevalence of hyper-polypharmacy was consistently highest among those 65+ years (about 40% prior to diagnosis and more than half post diagnosis), and it more than tripled in both younger age groups after diagnosis. The proportion of people without polypharmacy (<5 drugs) decreased after cancer diagnosis, and with increasing multimorbidity the proportion decreased both pre and post cancer diagnosis. The absolute decrease in the number of people not exposed to polypharmacy: for individuals without condition and those with one condition, the decrease was 23% and 21.7% (therefore around 22% more people were exposed to polypharmacy in general), but it decreased to 15.2% and actually increased by .2% for 2 and 3+ conditions. The prevalence of polypharmacy post cancer diagnosis was five times higher among those without any other chronic conditions and twice as high among those with one other chronic condition compared to prior to a cancer diagnosis respectively, but it remained stable for those with three or more conditions.

**Table 4.** Prevalence of Polypharmacy Within One Year Prior and Within One Year Post Cancer Diagnosis Overall and by Age, Sex, Multimorbidity, Cancer Type, and Cancer Stage.

Variables	Polypharmacy Prior to Diagnosis			Polypharmacy Post Diagnosis		
	No Polypharmacy (<5 drugs) (%)	Minor Polypharmacy (5-9 drugs) (%)	Hyper-Polypharmacy (10+ drugs) (%)	No Polypharmacy (<5 drugs) (%)	Minor Polypharmacy (5-9 drugs) (%)	Hyper-Polypharmacy (10+ drugs) (%)
Overall	54	20.9	25.1	43.3	18.9	37.8
Sex^a,b^						
Males	52	22.6	25.4	42	20	38.1
Females	56	19.3	24.7	44.6	17.9	37.5
Age^a,b^						
18-44 years	95.1	2.6	2.2	78.2	9.2	12.6
45-64 years	90.2	4.2	5.7	71	10.5	18.5
65+ years	24.4	34.6	41.1	20.2	25.8	54
Multimorbidity before cancer						
No condition^a,b^	94	5	1	71	14.7	14.3
1 condition^a,b^	80.4	15.3	4.4	58.7	19.7	21.6
2 conditions^a,b^	59	26.6	14.4	43.8	23	33.3
3+ conditions^a,b^	27.6	25.7	46.8	27.8	17.9	54.4
Cancer type^b^						
Breast cancer	67.5	17	15.6	44.9	20.9	34.2
Colon & rectum	49.4	24.1	26.5	36.1	21.1	42.8
Digestive system	44	22.6	33.5	36.2	17.1	46.8
Female genital	65.8	17	17.3	53.5	16.4	30.2
Hematological	50.6	21.5	27.9	42.3	17.2	40.6
Lung & bronchus	36.7	24.4	39	31.7	15.9	52.4
Prostate	55.3	24.8	19.9	47.5	24.8	27.7
Urinary system	45.3	23.4	31.3	37.2	20	42.8
Other	62.3	16.5	21.2	53.5	16	30.5
Cancer stage^b^						
Stage I	63.3	18	18.7	52.9	18.8	28.3
Stage II	57.8	22.4	19.8	43	22.6	34.4
Stage III	57.8	20.2	22	34.5	18.2	47.3
Stage IV	48.6	22.9	28.5	31.8	16.9	51.3
Unknown	49.1	21.1	29.9	45.9	18.1	36

^a^Statistically significant difference pre diagnosis (*P* < .0001).

^b^Statistically significant difference post diagnosis (*P* < .0001).

Polypharmacy status post cancer diagnosis differed significantly by type of cancer (*P* < .0001). There was a large difference in the proportion of persons with no polypharmacy between types of cancer; in persons with lung & bronchus, only 31.7% were not on polypharmacy but there were 53.5% for female genital cancer, a very large difference between types of cancer. The proportion of persons taking fewer than five medications steadily decreased with increasing stages of cancer, while hyper-polypharmacy steadily increased (*P* < .0001).

### Interaction Between Multimorbidity, Sex, Age, and Polypharmacy Post Cancer Diagnosis

**Figure 1.**
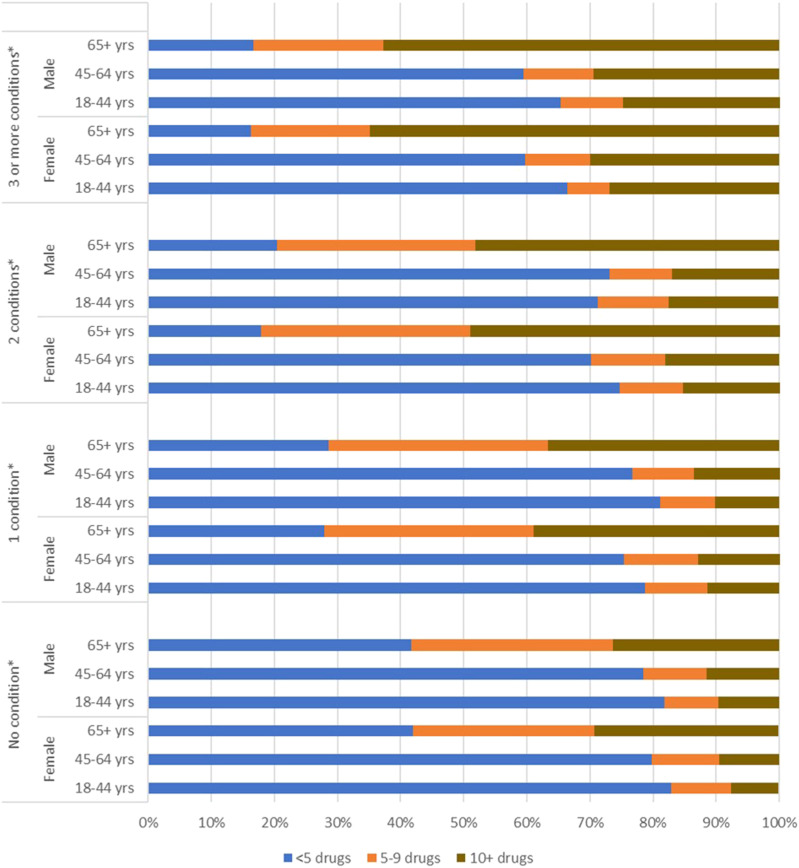
Prevalence of Polypharmacy, by Multimorbidity Level, Age and Sex Among Patients Diagnosed With Cancer, Ontario. ^*^P < .0001.

As shown on Figure 1, the association of polypharmacy post cancer diagnosis with sex and age was significant for each level of multimorbidity. The combination of old age and highest level of multimorbidity contributes to the highest prevalence of hyper-polypharmacy, regardless of sex, though in the older age group, the prevalence of hyper-polypharmacy remains higher for females across all levels of multimorbidity. The lowest prevalence of hyper-polypharmacy is seen in the youngest age group across levels of multimorbidity. However, the relationship with sex is less straightforward. Specifically, it appears that hyper-polypharmacy is lowest among females with no or two conditions, and among males with either one or three or more conditions.

These findings remain similar while considering type and stage of cancer. Table 5 shows how the interaction between multimorbidity and age impacts polypharmacy after a cancer diagnosis after adjusting for cancer type and stage of cancer. As the number of conditions and age increased the likelihood of being prescribed 5 or more drugs after cancer diagnosis increased, therefore, polypharmacy prevalence was impacted by multimorbidity level. The odds ratio (OR) for the interaction of 3+ conditions and being 65 years and older is 25.2, the highest risk of polypharmacy (5+ drugs). Whereas being 18-44 years with no chronic conditions had the lowest risk.

**Table 5.** Logistic Regression Model Assessing the Interaction Between Multimorbidity and Age on Polypharmacy (5+ Drugs) After Cancer Diagnosis (N = 193 047).

Variable	OR	99% CI
Sex (ref = female)		
Male*	1.12	1.08-1.16
Cancer type (ref = other)		
Breast*	1.59	1.50-1.68
Colon & rectum*	1.13	1.07-1.20
Digestive system (except colon)*	1.22	1.15-1.30
Female genital	1.01	.94-1.08
Hematological*	1.25	1.18-1.32
Lung and bronchus	.99	.94-1.05
Prostate*	.64	.60-.68
Urinary system*	1.28	1.20-1.38
Stage of cancer (ref = I)		
II*	1.59	1.51-1.68
III*	2.55	2.41-2.70
IV*	2.30	2.17-2.43
Unknown	.94	.89-.98
Multimorbidity and age group (ref = no condition and 18-44 years)		
no condition and 45-64 years	1.10	.99-1.22
no condition and 65 and older*	6.29	5.62-7.04
1 condition and 18-44 years	1.18	1.04-1.34
1 condition and 45-64 years*	1.29	1.17-1.42
1 condition and 65 and older*	11.7	10.60-12.95
2 conditions and 18-44 years*	1.59	1.37-1.85
2 conditions and 45-64 years*	1.68	1.52-1.85
2 conditions and 65 and older*	19.8	17.94-21.87
3+ conditions and 18-44 years*	2.62	2.21-3.12
3+ conditions and 45-64 years*	2.95	2.67-3.24
3+ conditions and 65 and older*	25.2	22.94-27.62

*P < .0001.

### Impact of Polypharmacy on Health Service Utilisation

Overall, respectively 10.7% and 21.3% of the study population were high users of the ER services and hospitalizations in the year following cancer diagnosis. Table 6 shows the relationship between polypharmacy pre-cancer diagnosis and health service utilization, controlling for all other factors (age, sex, multimorbidity, cancer type, cancer stage, number of chemotherapy drugs). Persons with hyper-polypharmacy were 41% (99% CI: 33%-51%) and 23% (17%-29%) more likely to be high users of ER services and hospitalizations, respectively, compared to those prescribed less than 5 drugs; taking 5-9 medications was not significantly different from taking <5 drugs. Male sex, increasing levels of multimorbidity and cancer stages, as well as higher numbers of chemotherapy drugs were positively associated with both high use of ER services and hospitalizations, whereas individuals of older age were less likely to be high users. Cancer related to the digestive system, prostate, and urinary system were also positively associated with high use.

**Table 6.** Logistic Regression Models on the Association Between Polypharmacy and Service Utilization.

	High users of ER (N = 193 047)		High users of hospital (N = 193 047)	
	OR	99% CI	OR	99% CI
Prior polypharmacy (ref ≤5)				
5-9 drugs	1.16*	1.09-1.24	1.03	.98-1.09
10 or more drugs	1.41*	1.33-1.51	1.23*	1.17-1.29
Sex (ref = female)				
Male	1.16*	1.11-1.22	1.21*	1.17-1.25
Age (ref = 18-44)				
45-64 years	.75*	.70-.81	.91*	.86-.97
65 and older	.48*	.44-.53	.74*	.69-.79
Multimorbidity (ref = none)				
1 condition	1.28*	1.18-1.38	1.08*	1.02-1.14
2 conditions	1.45*	1.34-1.56	1.16*	1.10-1.23
3+ conditions	1.77*	1.64-1.91	1.35*	1.28-1.43
Cancer type (ref = other)				
Breast	.92	.85-1.00	.46*	.43-.0.49
Colon & rectum	.98	.91-1.06	1.35*	1.28-1.43
Digestive system (except colon)	1.29*	1.20-1.40	1.62*	1.53-1.71
Female genital	1.10	1.00-1.22	1.10	1.02-1.18
Hematological	1.03	.95-1.11	1.05	.99-1.11
Lung and bronchus	1.02	.95-1.10	1.05	.99-1.11
Prostate	.73*	.66-.79	.28*	.26-.31
Urinary system	1.45*	1.33-1.59	1.52*	1.43-1.62
Stage of cancer (ref = I)				
II	1.25*	1.16-1.34	1.64*	1.55-1.75
III	1.50*	1.39-1.62	2.29*	2.16-2.43
IV	1.41*	1.31-1.52	2.54*	2.39-2.69
Unknown	1.01	.94-1.08	1.32*	1.25-1.39
Chemotherapy drugs (ref ≤5)				
5-9 drugs	2.28*	2.14-2.42	1.99*	1.89-2.09
10 or more drugs	3.00*	2.84-3.18	2.01*	1.91-2.11

*P < .0001.

## Discussion

The results of this study add to the body of literature demonstrating a relationship between polypharmacy (before and after cancer diagnosis) and older age and morbidity.^34^ It also adds to the body of literature by examining younger age groups, which are not often considered.

Polypharmacy has numerous definitions, and regardless of the definition the presence of multimorbidity often leads to multiple medications.^3^ The prevalence of polypharmacy was higher in males and older age; it also differed by levels of multimorbidity, which, combined with age had the largest impact. Adults 65+ years had the highest proportion of 3+ conditions, which explains the higher prevalence of polypharmacy in this age group. The observed increase in polypharmacy within and across multimorbidity levels by age is consistent with other general studies,^35,36^ and studies that included persons with cancer and multimorbidity.^37,38^ While comparable proportions of polypharmacy both across and within multimorbidity level were observed among males and females, regardless of age, other studies have shown variations in the prevalence between the sexes at different ages.^39,40^

The prevalence of polypharmacy was higher after a cancer diagnosis in all age groups; this is not surprising as pharmacotherapy is one of the most common treatment modalities for cancer management.^41^ However, it is crucial to point out that, consistent with the literature,^42^ the youngest age group was prescribed more chemotherapeutic drugs. As such, there was an absolute increase in polypharmacy in this group post cancer diagnosis. Further, older adults in particular may experience drug therapy related problems that require additional drugs as a form of management.^43^ However, older adults with cancer who are frail, have very high levels of multimorbidity, or other physiologic compromises are less likely prescribed chemotherapy than younger adults.^44,45^ Also, the increase in the number of drugs prescribed in younger adults can possibly be due to nonadherence issues. Nonadherence to drug therapy is common in young adults (18+ years) with cancer; studies report that it ranges from 27% to 60% in some types of cancer.^46,47^ Failure to comply with recommended drug regimens can result in worsening of disease that may result in additional drug therapy or alternative management in younger adults.

Presence of hyper-polypharmacy prior to cancer diagnosis was significantly associated with being a high user of ER services and hospitalizations one year post diagnosis. Polypharmacy can be considered a marker for an individual’s underlying health status.^48,49^ Other population-based studies showed that the risk of ER visits and hospitalizations increased with the number of drugs prescribed in persons with different cancer types.^50,51^ An adverse drug event can be a consequence of polypharmacy and is one of the main drug related reasons for ER visits and hospitalizations.^50,51^

Consistent with other studies, those with 3+ chronic conditions were more likely to be high users of ER services and hospitalizations. This study also showed that people 45-65 years and 65+ years were significantly less likely to be high users of ER services and hospitalizations than those aged 18-44. These findings contradict other study results^52,53^ regarding older age being associated with increased health service utilization. This could potentially be explained by the fact that older adults typically have longer length of hospital stay, and this study focused on extremely frequent use, rather than overall use. In fact, more older adults may use the services, and longer, but not as repetitively as younger adults.

This study’s results support current literature regarding the relationship between health service utilization and sex,^54^ types of cancer,^55^ advanced stages of cancer,^56^ and increased number of chemotherapy drugs.^57,58^ Moreover, we found that some types of cancer, advanced stages of cancer, and increased number of chemotherapy drugs were associated with a higher risk of being a high user of ER services and hospitalizations, similar to other studies.^55-57,59^ Certain cancer types and more advanced stages of cancer determine the frequency of health service utilization. For example, people with lung cancer have more symptom distress compared to other cancers which may result in more frequent health service utilization^59,60^ and more advanced stage cancer increases the risk of health service utilization due to their requirement for supportive management.^60,61^ Both the type and stage of cancer impact the number of drugs prescribed, including the type of chemotherapeutic agents which can potentiate the severity of drug therapy related problems. Studies also show that certain types of cancers that require high dose antineoplastic therapy may involve hospital admission,^62,63^ thus increasing the risk of health service utilization. Various determinants such as dosing frequency and required continuous infusions make the inpatient administration of chemotherapy the preferred practice for these persons.^64-68^ Chemotherapy regimens consisting of high doses can potentially cause toxicities and adverse drug events, and therefore hospital admission is recommended as best practice.^62,66^

This study is a population-based study using a cohort design with established methods, which was a major strength. As such it included all persons covered by the Ontario Drug Benefit program during the study period and also persons not covered, particularly all persons aged 65+ years, therefore minimizing selection bias. Most studies that assessed the prevalence of polypharmacy included mainly older adults with and without cancer; however, this study has extended the age of the population to include younger adults (18-64 years). We included all persons, those with ODB program coverage and those without coverage and therefore, the impact of polypharmacy was underestimated, a misclassification bias. Persons with no coverage were classified as having zero medications, this may not have been the case, they may have had private insurance coverage or paid out of pocket for their medications. According to the literature Canadians have their prescription drugs covered by private or public plans, with employer-sponsored drug benefits being a large source of drug coverage, about 60%.^69,70^ In 2012, public drug plans covered roughly 44% of drug costs, with the remainder being covered by private insurance plans and out of pocket.^70^ According to the Ontario Ministry of Health fiscal year report 2019-2020 more than 940 000 Ontarians 24 years and younger receive ODB program coverage.^71^

However, an important limitation is that only a small proportion of those 18-64 years is eligible to have their drugs covered by the Ontario Drug Benefit program. Besides, given the data sources, the study population included individuals who received care through the health care system of Ontario which may have also resulted in a selection bias.^72^ However, Ontario has a universal health coverable for its population and such bias should be minimized. Misclassification bias with regards to multimorbidity is also a possible limitation, given that the diagnosis and documentation of concomitant diseases may be based on physician practices and the reporting of diseases.^73^ However, if anything, the impact on the prevalence of polypharmacy would have been underestimated, as it is possible that some people deemed with no condition and their related medications should have been included in higher levels of multimorbidity. This study evaluated only the number of additional chronic conditions, which may be insufficient to understand the extent of polypharmacy related to disease severity among persons with cancer and multimorbidity. Being prescribed 10+ drugs was significantly associated with being a high user of ER services one year after cancer diagnosis (according to the literature increased number of drugs increases the risk of adverse drug events, and adverse drug events are one of the main drug related reasons for emergency room visits). Our study did not specifically study disease progression and whether it resulted in being a high user of ER services, however, it showed that persons with 3+ chronic conditions were more likely to be high users of ER services. The extent of polypharmacy related to disease severity or progression was not studied, though this may be somewhat captured by cancer stage, as disease may progress rapidly (within one year) for more severe cases. Also, we did not know the stage of cancer for about half of the sample. Though relevant for the impact on patients’ outcomes, inappropriate prescribing was beyond this paper and not considered in the study; this could be addressed in further work, as well as other possible additional confounders that were not available in the data.

## Conclusion

Given that the prevalence of multimorbidity is increasing across age groups, there is need for additional studies that delve further into the nature of polypharmacy – ie, appropriate vs inappropriate, to ensure not only better quality of life for individuals, but also more effective use of the health care system. While older adults were at increased risk for polypharmacy and multimorbidity, our study showed that these issues are also present in younger adults. The inclusion of a younger group in our study is important as it may inform age-specific recommendations and targeted actions, such as patient education. Based on our study findings, it is evident that involvement of a multidisciplinary team that includes the input of clinical pharmacists, is needed to monitor and adjust medication lists to reduce or control polypharmacy, and thus ensure optimal outcomes in adults, both young and old, with cancer and multimorbidity.

## Supplemental Material

Supplemental Material - Understanding the Extent of Polypharmacy and its Association With Health Service Utilization Among Persons With Cancer and Multimorbidity: A Population-Based Retrospective Cohort Study in Ontario, Canada Click here for additional data file. Supplementary Material for Understanding the Extent of Polypharmacy and its Association With Health Service Utilization Among Persons With Cancer and Multimorbidity: A Population-Based Retrospective Cohort Study in Ontario, Canada by Tamara Dean, Anna Koné, Lynn Martin, Joshua Armstrong, and Caroline Sirois in Journal of Pharmacy Practice.
