# Supplementary material for: Understanding the Extent of Polypharmacy and its Association With Health Service Utilization Among Persons With Cancer and Multimorbidity: A Population-Based Retrospective Cohort Study in Ontario, Canada
Source: J Pharm Pract. 2022 Jul 21;37(1):35–46. doi: 10.1177/08971900221117105 (PMC10804697; doi:10.1177/08971900221117105)
Supplement: Supplemental Material - Understanding the Extent of Polypharmacy and its Association With Health Service Utilization Among Persons With Cancer and Multimorbidity: A Population-Based Retrospective Cohort Study in Ontario, Canada [file sj-pdf-1-jpp-10.1177_08971900221117105.pdf]

Appendix A: List of ICD codes defining cancer and the 17 selected conditions (from Kone et al: *Can J Public Health* **112,** 737–747 (2021). https://doi.org/10.17269/s41997-021-00474-y)

**Condition [reference for validated algorithm]**	**ICD 9 / OHIP**	**ICD 10**	**Drug Subclass (ODB)***
Acute Myocardial Infarction (AMI) [1]	410	I21	
Osteo- and other Arthritis:			
(A) Osteoarthritis	715	M15-M19	
(B) Other Arthritis (includes Synovitis, Fibrositis, Connective tissue disorders, Ankylosing spondylitis, Gout Traumatic arthritis, pyogenic arthritis, Joint derangement, Dupuytren's contracture, Other MSK disorders)	727, 729, 710, 720, 274, 716, 711, 718, 728, 739	M00-M03, M07, M10, M11-M14, M20-M25, M30-M36, M65-M79	
Arthritis - Rheumatoid arthritis [2]	714	M05-M06	
Asthma [3]	493	J45, J46	
(all) Cancers	140-239	C00-C26, C30-C44, C45-C97	
Cardiac Arrhythmia	427 (OHIP) / 427.3 (DAD)	I48.0, I48.1	
Congestive Heart Failure [4]	428	I500, I501, I509	
Chronic Obstructive Pulmonary Disease [5]	491, 492, 496	J41, J42, J43, J44	
Coronary syndrome (excluding AMI)	411-414	I20, I22-I25	
Dementia [6]	290, 331 (OHIP) / 046.1, 290.0, 290.1, 290.2, 290.3, 290.4, 294, 331.0, 331.1, 331.5, F331.82† (DAD)	F00, F01, F02, F03, G30	Cholinesterase Inhibitors
Diabetes [7]	250	E10, E11, E13, E14	Oral anti-glycemics, Insulin, Anti-diabetic agents: Misc
Hypertension [8]	401, 402, 403,	I10, I11, I12, I13, I15	

	404, 405	
(Other) Mental health condition	291, 292, 295, 297, 298, 299, 301, 302, 303, 304, 305, 306, 307, 313, 314, 315, 319	F04, F050, F058, F059, F060, F061, F062, F063, F064, F07, F08, F10, F11, F12, F13, F14, F15, F16, F17, F18, F19, F20, F21, F22, F23, F24, F25, F26, F27, F28, F29, F340, F35, F36, F37, F430, F439, F453, F454, F458, F46, F47, F49, F50, F51, F52, F531, F538, F539, F54, F55, F56, F57, F58, F59, F60, F61, F62, F63, F64, F65, F66, F67, F681, F688, F69, F70, F71, F72, F73, F74, F75, F76, F77, F78, F79, F80, F81, F82, F83, F84, F85, F86, F87, F88, F89, F90, F91, F92, F931, F932, F933, F938, F939, F94, F95, F96, F97, F98
Mood, anxiety, depression and other nonpsychotic disorders	296, 300, 309, 311	F30, F31, F32, F33, F34 (excl. F34.0), F38, F39, F40, F41, F42, F43.1, F43.2, F43.8, F44, F45.0, F45.1, F45.2, F48, F53.0, F68.0, F93.0, F99
Osteoporosis	733	M81, M82
Renal disease	403, 404, 584, 585, 586, v451	N17, N18, N19, T82.4, Z49.2, Z99.2
Stroke (excluding transient ischemic attack)	430, 431, 432, 434, 436	I60-I64

References:

1. Austin PC, Daly PA, Tu JV. A multicenter study of the coding accuracy of hospital discharge administrative data for patients admitted to cardiac care units in Ontario. American Heart Journal 2002;144:290–6.
2. Widdifield J, Bernatsky S, Paterson JM, Tu K, Ng R, Thorne JC, Pope JE, Bombardier C. Accuracy of Canadian health administrative databases in identifying patients with rheumatoid arthritis: a validation study using the medical records of rheumatologists. Arthritis Care Res 2013; 65(10): 1582-1591.
3. Gershon AS, Wang C, Guan J, Vasilevska-Ristovska J, Cicutto L, To T. Identifying patients with physician-diagnosed asthma in health administrative databases. Can Respir J 2009;16:183–8.

4. Schultz SE, Rothwell DM, Chen Z, Tu K. Identifying cases of congestive heart failure from administrative data: a validation study using primary care patient records. Chronic Diseases and Injuries in Canada 2013;33:160–6.
5. Gershon AS, Wang C, Guan J, Vasilevska-Ristovska J, Cicutto L, To T. Identifying Individuals with Physician Diagnosed COPD in Health Administrative Databases. Copd 2009;6:388–94.
6. Jaakkimainen RL, Bronskill SE, Tierney MC, Herrmann N, Green D, Young J, et al. Identification of Physician-Diagnosed Alzheimer's Disease and Related Dementias in Population-Based Administrative Data: A Validation Study Using Family Physicians' Electronic Medical Records. J Alzheimers Dis. 2016 Aug 10;54(1):337–49
7. Hux JE, Ivis F, Flintoft V, Bica A. Diabetes in Ontario: Determination of prevalence and incidence using a validated administrative data algorithm. Diabetes Care 2002;25:512–6.
8. Tu K, Campbell NR, Chen Z-L, Cauch-Dudek KJ, McAlister FA. Accuracy of administrative databases in identifying patients with hypertension. Open Med 2007;1:e18–26.
